# Force profile assessment of direct-printed aligners versus thermoformed aligners and the effects of non-engaged surface patterns

**DOI:** 10.1186/s40510-022-00443-2

**Published:** 2022-11-29

**Authors:** Evan Hertan, Julie McCray, Brent Bankhead, Ki Beom Kim

**Affiliations:** Department of Orthodontics, Saint Louis University, 3320 Rutger Street, Saint Louis, MO 63104 USA

## Abstract

**Background:**

The purpose of the study was to measure the forces delivered by direct-printed aligners (DPA) in the vertical dimension and compare the force profile with traditional thermoformed aligners (TFA) and to investigate the impact of non-engaged surface patterns to the properties of DPA and TFA.

**Methods:**

A force-measuring appliance was fabricated capable of displacing the aligner in 0.10 mm increments and measuring the resultant force. Polyethylene terephthalate glycol (ATMOS 0.030″ American Orthodontics) and TC-85DAC resin (Graphy Inc) were used to create TFA and DPA, respectively. Aligners were temperature-controlled prior to and during testing to simulate the oral environment. The resultant forces from displacements ranging from 0.10 to 0.30 mm were measured.

**Results:**

At intraoral temperatures, DPA demonstrated significantly less force than TFA. TFA demonstrated a substantial statistically significant increase in force with each 0.10 mm increase in vertical displacement. DPA demonstrated a much more consistent force profile across the range of displacements. The effects of surface patterns in both DPA and TFA were generally a decrease in force. Statistical significance of surface patterns was detected for TFA at displacements of 0.30 mm and greater and significant for DPA only at a displacement of 0.10 mm. Surface patterns in both DPA and the TFA did not show any statistical difference when assessing force proprieties.

**Conclusions:**

Forces delivered by aligners in the vertical dimension by DPA are more consistent and of lower magnitude than those of TFA aligners. Surface patterns were not capable of altering the force properties of both DPA and TFA.

## Background

New technological developments and market demands have rapidly increased the availability and affordability of intraoral scanners and 3D printers. These technological advancements combined with the market demand for aesthetic treatment options have driven a surge in the use of clear aligners for orthodontic tooth movement [1, 2]. Clear Aligner treatment utilizing 3D printing technology has been limited to printing 3D models with staged tooth movements and subsequently thermoforming plastic sheets to create the desired aligners. The prospect of direct 3D printing of aligners themselves offers to usher in an era of innovation. Specifically, the direct 3D printing of aligners offers the opportunity to control material dimensions, structure, and properties more directly [3, 4]. Furthermore, direct 3D printing of aligners offers the promise of reduced waste [5], improved turnaround time, and an era of on-demand clear aligner treatment. [4, 6, 7]

Direct-printed aligners (DPA) in contrast to traditional thermoformed aligners (TFA) offer to usher in a new world of opportunities and possibilities to control tooth movements through novel techniques. Specifically, the creation of different thicknesses throughout the appliance or utilization of discrete pressure points or other patterns and surface textures or shapes may be able to generate a couple or improved biomechanics thereby removing or minimizing the need for attachments [4, 8]. The potential promise of 3D surface patterns, shapes, and techniques may be able to fundamentally modify the elasticity or rigidity of aligners in order to deliver improved biomechanics and expedite treatment [4]. The purpose of the study was to measure the forces delivered by DPA in the vertical dimension and compare the force profile with TFA and to investigate the impact of non-engaged surface patterns to the properties of DPA and TFA.

## Methods

### Sample preparation

**Fig. 1:**
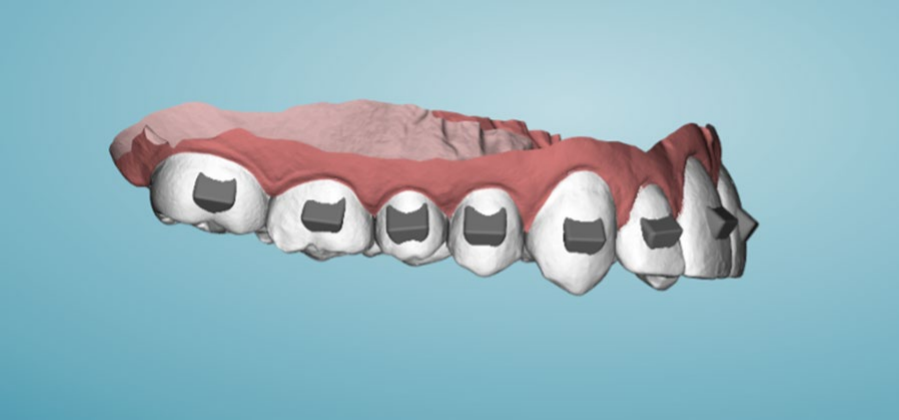
Design of the YA Master Model with attachments as designed in uDesign

A master scan of a maxillary arch was captured utilizing a Trios Scanner (3Shape, Copenhagen, Denmark), and exported into uDesign 6.0 software (uLab Systems Inc., San Mateo, CA, USA). Two digital master models were produced: one had no attachments (NA), just the trimmed maxillary model while the other had attachments (rectangular, gingivally beveled horizontal attachments with a depth of 2.7 mm, a height of 4.2 mm, and a width of 4.0 mm (Fig. 1)) on all the maxillary teeth (YA). Four master models (2 NA & 2 YA) were printed with Sprint Ray Pro DLP Printer (SprintRay, Los Angeles, CA, USA) at 100 µm-layer thickness. SprintRay Die and Model Gray II photo-initiated methacrylate resin with a flexural modulus of 2650 MPa and a Flexural strength of 91.5 MPA was used for master model 3D printing fabrication.

### Thermoformed aligner (TFA) fabrication

Models were processed following the resin manufacturer recommendations. They were cured using the SprintRay Pro Cure (SprintRay, Los Angeles, CA, USA). ATMOS thermoforming plastic 125 mm round sheets with 0.030″ thickness (American Orthodontics, Sheboygan, WI, USA) were thermoformed over the master models utilizing a Biostar (Scheu-Dental GmbH, Iserlohn, Germany) pressurized thermoforming machine per manufacturer recommendations. A total of 20 thermoformed aligners were created, 10 of the TFA-NA and 10 of the TFA-YA.

### Direct-printed aligner (DPA) fabrication

DPA sample was fabricated utilizing the same digital NA and YA master models with uDesign 6.0 beta software. Aligners were digitally trimmed to approximately 1 mm past the gingival margin. 0.50 mm thickness and 0.05 mm offset of aligner from model were utilized. Two master aligner files were created with this method: DPA with no attachments (DPA-NA) and DPA with attachments (DPA-YA) were fabricated and exported as STL Files. The DPA master files were then imported into Uniz Software (Uniz, San Diego, CA, USA), rotated to -110 degrees and supports generated. DPA Aligners were printed on Sprint Ray Pro95 printer at 100 µm-layer thickness. Graphy Tera Harz TC-85DAC resin was used for printing (Graphy Inc, Seoul, Korea). The properties of the printed resin are described by the company as Shore Hardness (D) > 85, Flexural strength > 65 MPa, Flexural Modulus > 1500 MPa.

DPA with intact supports were removed from the printer build plate and placed in a centrifuge for 3 min to remove uncured resin. The aligner was then removed from the supportive scaffolding with finger pressure. Aligners were cured in a Cure M machine (Graphy Inc, Seoul, Korea). Aligners were cured for 35 min with nitrogen gas, then submerged in glycerin and cured without nitrogen gas for an additional 35 min. A total of 20 DPA aligners were created, 10 of the DPA-NA and 10 of the DPA-YA.

### Test model preparation and fabrication

**Fig. 2:**
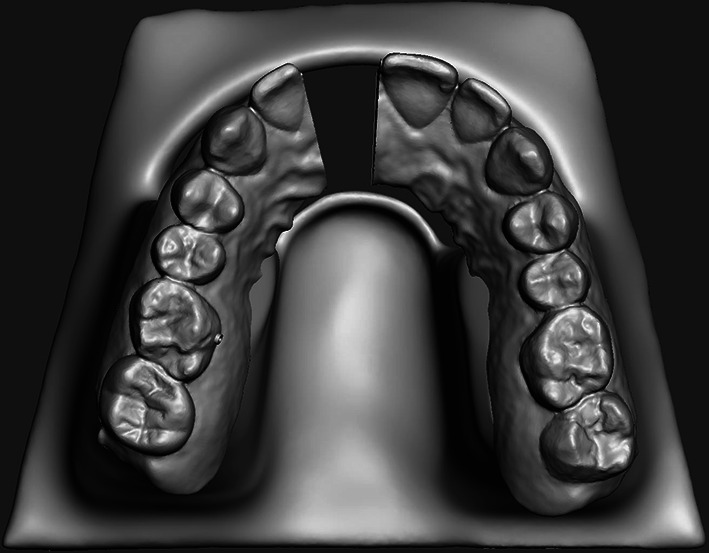
Design of the test model, note no attachments are present on the model

The test model was created by importing the master digital NA file exported into MeshMixer (Autodesk, San Rafael, CA, USA) where the model was segmented to remove UR1. The model was supported vertically to provide strength and clearance for materials testing (Fig. 2). The test model was printed with a Uniz Slash-C LCD 3D printer (Uniz, San Diego, CA, USA) utilizing AnyCubic Clear 3D Resin (AnyCubic, Shenzhen, China). The manufacturer reported resin properties are a shore hardness (D) of 79, tensile strength of 23.4 MPa and elongation of 14.2%.

### Measurement method

**Fig. 3:**
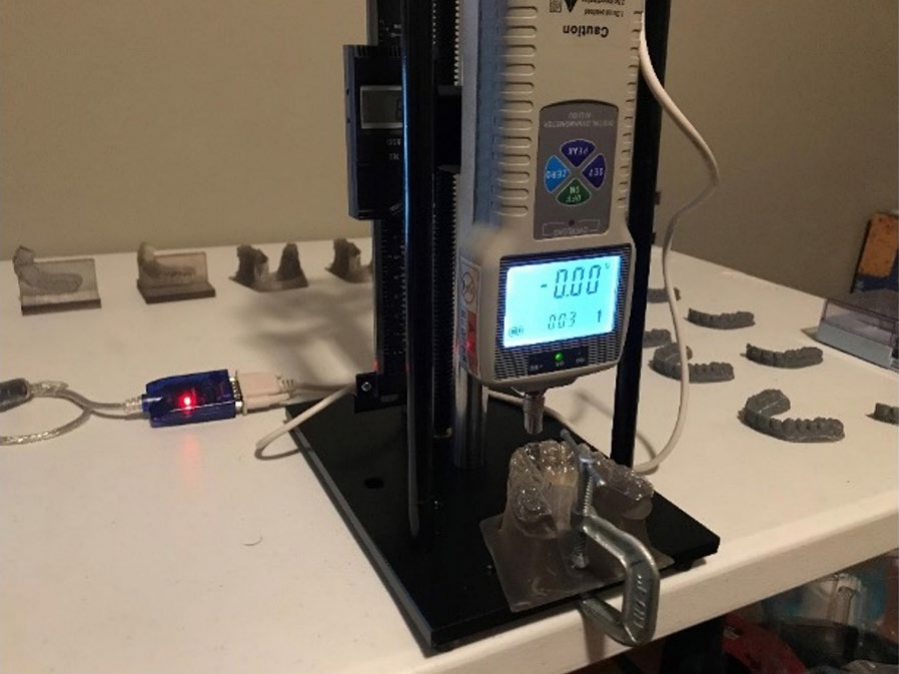
Experimental Test Stand with dynamometer and integrated caliper prior to initializing displacement test of TFA aligner on the test model

A hand wheel operated manual force test stand with integrated digital caliper with mm resolution to 0.01 mm was paired with a ZP-50 digital force gauge (Baoshishan, Shenzhen, China) with resolution to 0.01 N. Calibration of the ZP-50 dynamometer was verified with a handheld Correx dynamometer (Haag-Streit Diagnostics, Köniz, Switzerland). The ZP-50 dynamometer was secured to the test stand in compression test mode. The selected test model was secured to the baseplate of the test stand utilizing a standard mini c-clamp (Fig. 3).

Given the temperature-sensitive shape memory properties of DPA, it was necessary to simulate the oral environment. Aligners were heated to body temperature (97.5 F) for a minimum of 5 min prior to testing by placement of each aligner in an individual water-filled bag (30–60 ml) in a temperature-controlled water bath. To further maintain the intraoral simulated temperature environment, a ceramic positive thermal coefficient heater was used.

The newton meter was lowered incrementally until a force was read on the digital force meter after placing each aligner to the test model. The meter was then raised until the force equaled zero. This process was repeated three times for each sample. The digital caliper was then zeroed, and the aligner was compressed with vertical compression on external incisal edge of the missing UR1. Compression occurred until a displacement of 0.10 mm in the gingival direction and then peak N reading was recorded, a timer was then set and at 20 s, the N reading was recorded, compression then continued to 0.20 mm displacement with a subsequent peak N recording and a further N recording after 20 s of force stabilization. This process continued until 0.30 mm displacement. A total of 40 aligners were tested in this manner on the test model, 10 DPA-NA, 10 DPA-YA, 10 TFA-NA, and 10 TFA-YA. All recorded data indicated the tested aligner number for quality assurance and appropriate statistical analysis.

### Statistical methodology

Dynamometer readings were captured at each respective displacement. Readings were captured for peak force (N) and stabilized force (N).

All analyses were conducted using SAS version 9.3 (SAS Inc, Cary, NC) and the level of significance (α) was set to 0.05. Wilcoxon rank-sum test (nonparametric) was performed to compare the peak force and stabilized force among DPA and TFA with and without attachments.

## Results

### Force assessment

**Fig. 4:**
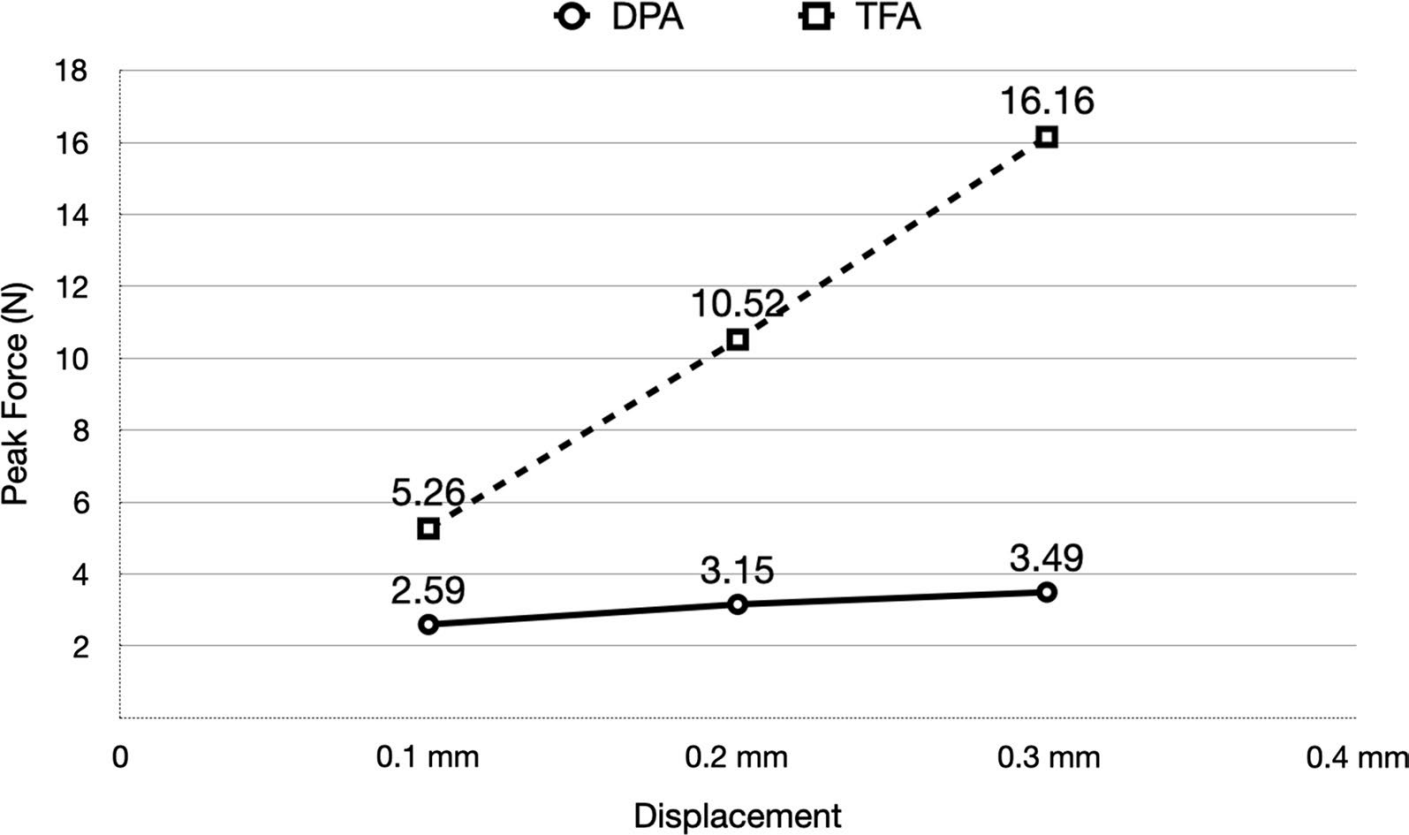
Comparison of Peak Forces of TFA versus DPA

The median stabilized forces demonstrated by TFA in response to 0.10–0.30 mm displacements ranged from 4.60 to 15.30 N. The median peak force demonstrated by TFA in response to 0.10–0.30 mm displacements ranged from 5.11 to 16.26 N (Fig. 4, Table 1).

**Table 1:** Comparing TFA-NA (no attachments) and TFA-YA (with attachments)

Displacement	Unit (N)	TFA-NA		TFA-YA		*p* value
		Mean ± SD	Median	Mean ± SD	Median	
0.10 mm	Peak force	5.26 ± 0.51	5.11	5.13 ± 0.89	5.34	0.94
	Stabilized force	4.73 ± 0.50	4.60	4.6 ± 0.84	4.74	0.97
0.20 mm	Peak force	10.52 ± 0.69	10.52	10.37 ± 1.21	10.39	0.82
	Stabilized force	9.77 ± 0.76	9.68	9.60 ± 1.18	9.75	0.94
0.30 mm	Peak force	16.16 ± 0.71	16.10	15.85 ± 1.36	16.26	0.94
	Stabilized force	15.04 ± 0.8	14.89	14.84 ± 1.48	15.30	0.55

**Fig. 5:**
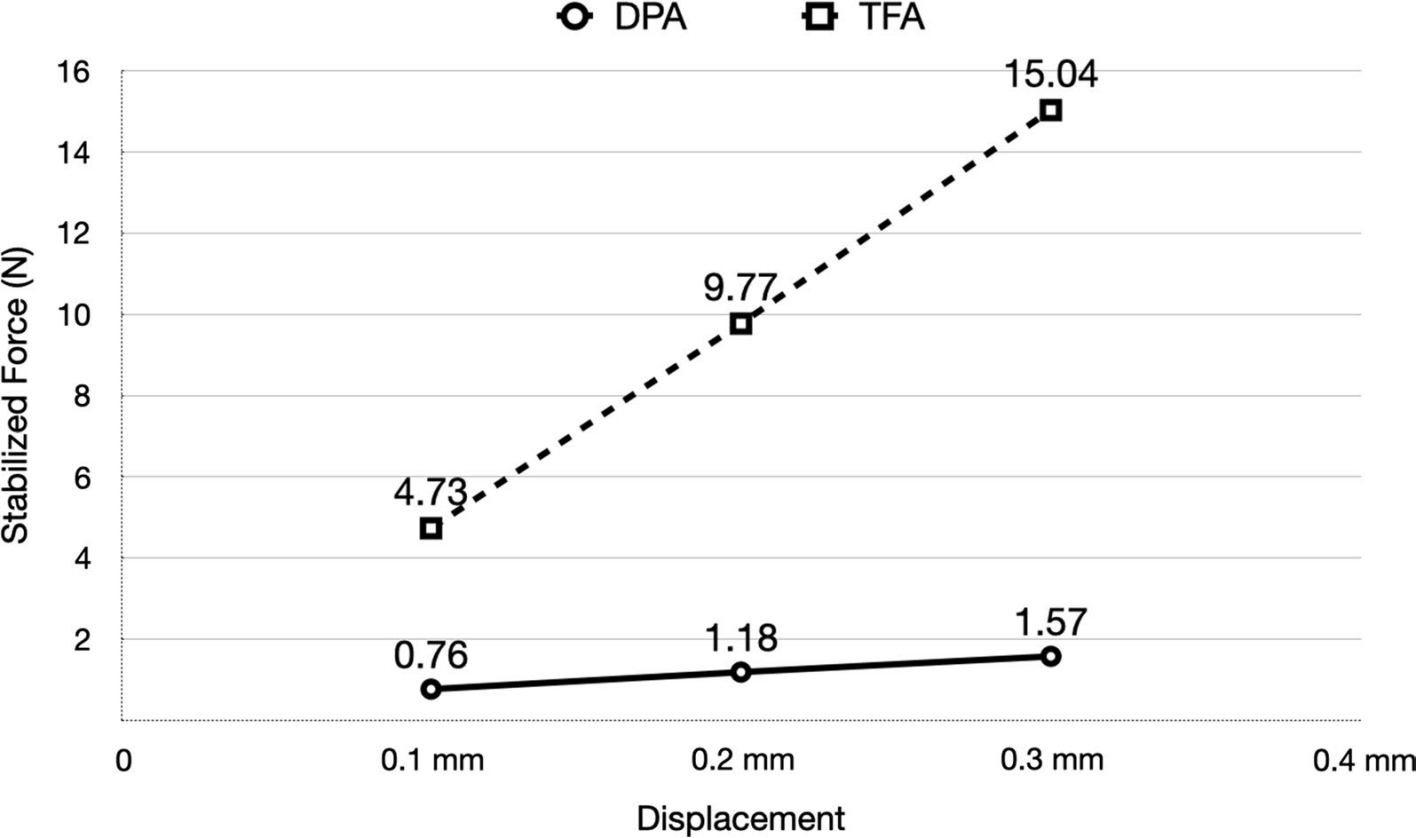
Comparison of Stabilized Forces of TFA versus DPA

The median stabilized forces that were demonstrated by DPA in response to 0.10—0.30 mm displacements ranged from 0.73 to 1.69 N. The median peak force demonstrated by DPA in response to 0.10–0.30 mm displacements ranged from 2.44 to 3.87 N (Fig. 5, Table 2).

**Table 2:** Comparing DPA-NA (no attachments) and DPA-YA (with attachments)

Displacement	Unit (N)	DPA-NA		DPA-YA		p value
		Mean ± SD	Median	Mean ± SD	Median	
0.10 mm	Peak force	2.59 ± 0.62	2.44	2.77 ± 0.60	2.65	0.45
	Stabilized force	0.76 ± 0.18	0.73	0.81 ± 0.21	0.79	0.65
0.20 mm	Peak force	3.15 ± 0.65	3.18	3.58 ± 0.51	3.52	0.14
	Stabilized force	1.18 ± 0.27	1.19	1.33 ± 0.23	1.26	0.15
0.30 mm	Peak force	3.49 ± 0.71	3.48	4.04 ± 0.67	3.87	0.08
	Stabilized force	1.57 ± 0.37	1.52	1.78 ± 0.39	1.69	0.24

DPA demonstrated significantly less force than TFA. TFA demonstrated a substantial statistically significant increase in force with each 0.10 mm increase in vertical displacement (Table 3).

**Table 3:** Comparing DPA and TFA

Displacement	Unit (N)	DPA				TFA				*p* value
		Mean ± SD	Median	Lower quartile	Upper quartile	Mean ± SD	Median	Lower quartile	Upper Quartile	
0.10 mm	Peak force	2.59 ± 0.62	2.44	2.25	3.12	5.26 ± 0.51	5.11	4.88	5.58	< .0001
	Stabilized force	0.76 ± 0.18	0.73	0.67	0.88	4.73 ± 0.50	4.60	4.30	5.13	< .0001
0.20 mm	Peak force	3.15 ± 0.65	3.18	3.01	3.82	10.52 ± 0.69	10.52	9.91	11.03	< .0001
	Stabilized force	1.18 ± 0.27	1.19	1.10	1.35	9.77 ± 0.76	9.68	9.14	10.21	< .0001
0.30 mm	Peak force	3.49 ± 0.71	3.48	3.29	4.18	16.16 ± 0.71	16.10	15.64	16.51	< .0001
	Stabilized force	1.57 ± 0.37	1.52	1.42	1.9	15.04 ± 0.8	14.89	14.41	15.54	< .0001

### The effect of unsupported attachments

TFA-YA did not show any statistically significant differences in comparison with TFA-NA in peak force. There were no statistically significant differences in stabilized force between TFA-YA and TFA-NA. There were no obvious trends or differences between TFA-YA and TFA-NA for 0.10–0.30 mm displacement.

DPA-YA did not show any statistically significant differences in comparison with DPA-NA in peak force. There were no statistically significant differences in stabilized force between DPA-YA and DPA-NA. DPA-YA generally delivered a stronger median force than DPA-NA, though this finding was not statistically significant.

## Discussion

TFA has been used for some decades [9]. Even with good clinical outcomes, its accuracy not always follows what was initially planned [10, 11]. The fabrication of aligners brings features that can change its geometrical proprieties and consequently, the biomechanical behavior and forces characteristics [10]. Different studies are available demonstrating the force behavior of TFA in a series of movements [12–15]. One study reported that initial force created by TFA can demonstrate 1–15 N. [12] Another study evaluating the forces applied on a central incisor when a labiopalatal body movement is projected, demonstrated that those forces can reach about 8.37 N. [13] Barbagallo et al. utilized a novel pressure-sensitive film to determine the force applied by an aligner in vivo. The amount of force with 0.80 mm thickness aligner on a maxillary premolar programmed with 0.5 mm of buccal tipping was 5.12 N. [14] Hahn et al. found that the forces had a higher magnitude than they were expected to be [16]. Proffit suggested that ideal orthodontic movement forces ranges from 10 to 120 g (0.10 to 1.18 N) [17]. Even though a systematic review demonstrated that there isn’t an article yet that can provide this exact data [18], the accepted clinical practiced in orthodontics remains the utilization of light forces as recommended by Proffit to minimize excessive hyalinization [17]. The current study showed that the median stabilized and peak force in displacements 0.3 mm with TFA reached 14.89 N and 16.1 N, respectively, a force profile was much higher than previous suggested [17, 19–21].

The force profile delivered by DPA was significantly lower than the ones demonstrated by TFA (Table 3) The median stabilized force delivered by DPA ranged from 0.73 N at 0.10 mm displacement to 1.52 N at 0.30 mm displacement.

Comparing the difference between the peak and stabilized force levels, DPA showed a larger force decay than TFA. Lee et al. also reported a similar result with the thermo-mechanical cycle property test that DPA showed a much bigger stress relaxation compared to TFA [22]. When comparing peak force, DPA showed 77% less force than TFA, while on stabilized force it was even more significant, reaching almost 90% less force. The literature does not show any data that can be used to compare our results with other studies on DPA, but current findings suggest that the forces delivered by DPA appear to be more aligned to the biomechanically desired levels recommended, delivering a more consistent force profile [17, 21]. In a sense, DPA could be considered analogous to NiTi wires delivering gentle consistent forces over a range of displacements.

The effects of attachments on the force delivered by aligners and retention have been extensively studied [10, 23–26]. However, the effects of attachments on the rigidity, flexibility, elasticity are not reported in the literature. One goal of the present study was to investigate how surface patterns can cause an effect on the forces of both TFA and DPA. At first, we hypothesized that surface patters such as unfilled attachments could demonstrate the ability to modify the mechanical force properties of the aligners, but based on our results, the null hypothesis could not be confirmed. When considering force peak and force stabilization in DPAs and TFA, and comparing between groups with or without attachments, no statistical significance could be found. An important note relevant to the experiment methodology is the fact that the spaces between the aligner and the tooth could serve as a stress break to increase flexibility, and that a measured increase or decrease in flexibility with attachments, while meaningful in a materials science aspect, may not translate to clinical significance. Further research is necessary to explore surface pattern options with a focus on direct-printed aligners and better harness their full potential.

Limitations to the current methodology include the lack of PDL in the experimental teeth; thus, the force generated may be of higher magnitude as compared with what would normally be expected in a system where all teeth have degrees of freedom corresponding to the PDL space. Furthermore, when the aligner is compressed onto the teeth clinically, there may be over-compression followed by a release. Additional limitations include the ability of the aligner to retain a tooth in question and the effect of this on the force profile.

Even with the stated limitations, the current data can still be considered significant. Confirming this, a systematic review analyzing forces and moments on aligners affirmed that tooth movements can be simulated in an effective manner in the in vitro environment [25]. Our findings are of important clinical relevance, as we demonstrate for the first time, that DPA delivers adequate amount of forces during on an extrusion movement. Additional studies are required to investigate stress relaxation behavior over time in the oral environment.

## Conclusions

Direct-printed aligners can deliver biologically compatible forces for orthodontic tooth movement in an in vitro setting. In contrast to thermoformed aligners, the forces delivered by direct-printed aligners may demonstrate improved ability to deliver forces within traditionally accepted range of optimum forces for tooth movement. The study demonstrates that surface pattern did not alter the force profile of aligners. Further investigation of surface patterns, ribbing, and other features in direct print aligners offers a new realm of opportunity in clear aligner research.

## Data Availability

The datasets used and/or analyzed during the current study are available from the corresponding author on reasonable request.
